# Plant direct author stories

**DOI:** 10.1002/pld3.176

**Published:** 2019-10-24

**Authors:** Ivan Baxter

Our journal's mission is to help our community by giving them an easier, faster way to get all their science out. Authors are choosing to publish in *Plant Direct* for different reasons—whether it is to add their first publication to their CV, to share initial findings that could be valuable to others working on similar research, or their paper has been referred from *Plant Cell, Plant Physiology* or *The Plant Journal,* and they agreed to transfer their manuscript and the reviews to save time.

We are excited to share the experiences of three investigators who have recently published in *Plant Direct*. Thank you Alexis Maizel, Elizabeth Haswell, and Joshua R. Widhalm for sharing your author stories with us and the *Plant Direct* community! We hope their stories inspire you and that you consider *Plant Direct* is as the right home for your next paper.

## ALEXIS MAIZEL

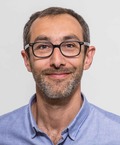

### What does your laboratory work on?

My laboratory studies the mechanisms of lateral root morphogenesis in Arabidopsis. We combine molecular genetics, cell biology, and microscopy to understand how these new roots are robustly formed.

### Tell us about the key findings from your recent article in *Plant Direct*

This article links to another interest of my laboratory: The role played the trans‐acting small interfering RNAs (ta‐siRNAs) in controlling root growth. The production of such ta‐siRNAs from their TAS3 precursors is triggered by the microRNA miR390. In this article, we report that miR390 is expressed in the transit‐amplifying compartment of the root meristem where it modulates response to exogenous auxin. The expression of miR390 in this region of the meristem depends on ARF5/MONOPTEROUS.

### How did you get the idea to do this study?

Comparatively, we know a lot about what miRNAs control but little about how they are themselves transcriptionally controlled. Nothing was known about the cis‐regulatory elements that govern the precise expression of miR390 in the root meristem and the lateral root primordia. We decided to map the promoter to test whether distinct enhancers could be identified.

### What do you plan to do next, based on these current findings?

Better characterize the gene regulatory network at play in the basal meristem.

### How was your experience with publishing in *Plant Direct*?

The manuscript had been submitted to *The Plant Cell*. The postreview decision made clear that although solid, the story would be a better fit for *Plant Direct*. The transfer of the manuscript and reviews was seamless and a decision to publish reached within days. It was a very efficient and professional process.

READ THE STUDY

Dastidar, M. G.
, 
Scarpa, A.
, 
Mägele, I.
, 
Ruiz‐Duarte, P.
, 
von Born, P.
, 
Bald, L.
, … 
Maizel, A.
 (2019). ARF5/MONOPTEROS directly regulates miR390 expression in the *Arabidopsis thaliana* primary root meristem. Plant Direct, 3, 1–12. 10.1002/pld3.116
PMC650884731245759

## ELIZABETH HASWELL

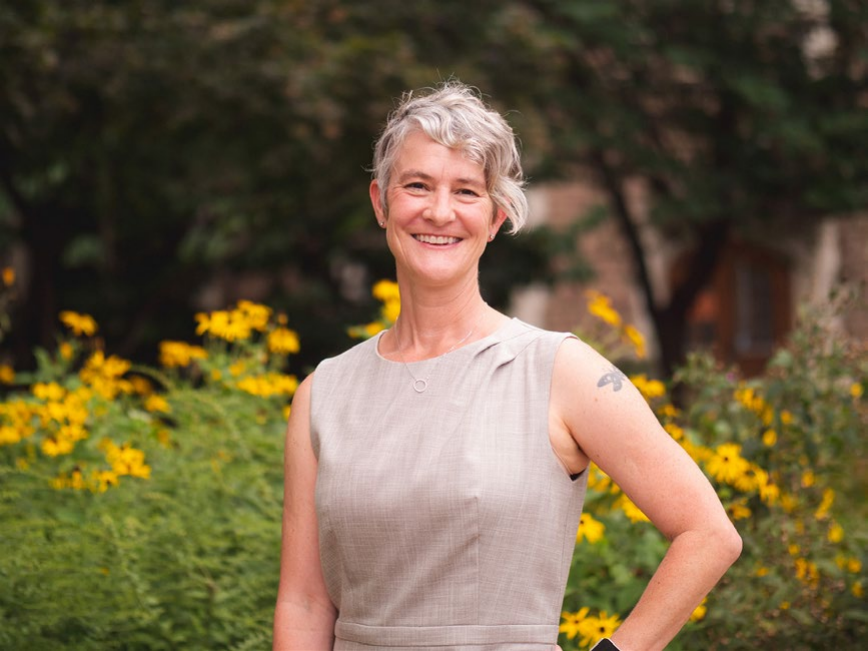

### What does your laboratory work on?

My group studies the cellular and molecular mechanisms by which plants sense and response to mechanical stimuli. In particular, we focus on a class of molecular mechanoreceptors called mechanosensitive ion channels.

### Tell us about the key findings from your recent article in *Plant Direct*

This project was meant to be a straightforward study of genetic and protein–protein interactions between three mechanosensitive ion channels from Arabidopsis thaliana, two that localize to the chloroplast, and one that localizes to the mitochondria. Things turned out to be a bit more complicated than we thought, and it turns out that loss of the mitochondrial channel exacerbates some phenotypes associated with loss of the chloroplast channels; but ameliorates others.

### Why did you choose to publish in *Plant Direct*?

For the most part, this paper comprises the senior thesis of Wash U undergraduate Josephine Lee. She worked in the laboratory for a few semesters for credit, and in the summer of 2015 was awarded an ASPB SURF to continue the project. The following semester she wrote everything up for her senior thesis. When I saw it, I realized that she'd collected some interesting results, and done so exceptionally carefully. We only had to make a few small changes to prepare her figures for publication! Josephine was really invested in published her work, both to share it with the field and to have a publication on her CV when she applies to graduate school. However, the complicated nature of her results would have made it difficult to publish in many journals without a lot of additional study. We chose *Plant Direct* for its focus on sound science rather than novelty or perceived impact.

READ THE STUDY

Lee, J. S.
, 
Wilson, M. E.
, 
Richardson, R. A.
, & 
Haswell, E. S.
 (2019). Genetic and physical interactions between the organellar mechanosensitive ion channel homologs MSL1, MSL2, and MSL3 reveal a role for inter‐organellar communication in plant development. Plant Direct, 3, 1–10. 10.1002/pld3.124
PMC650883131245767

## JOSHUA R. WIDHALM

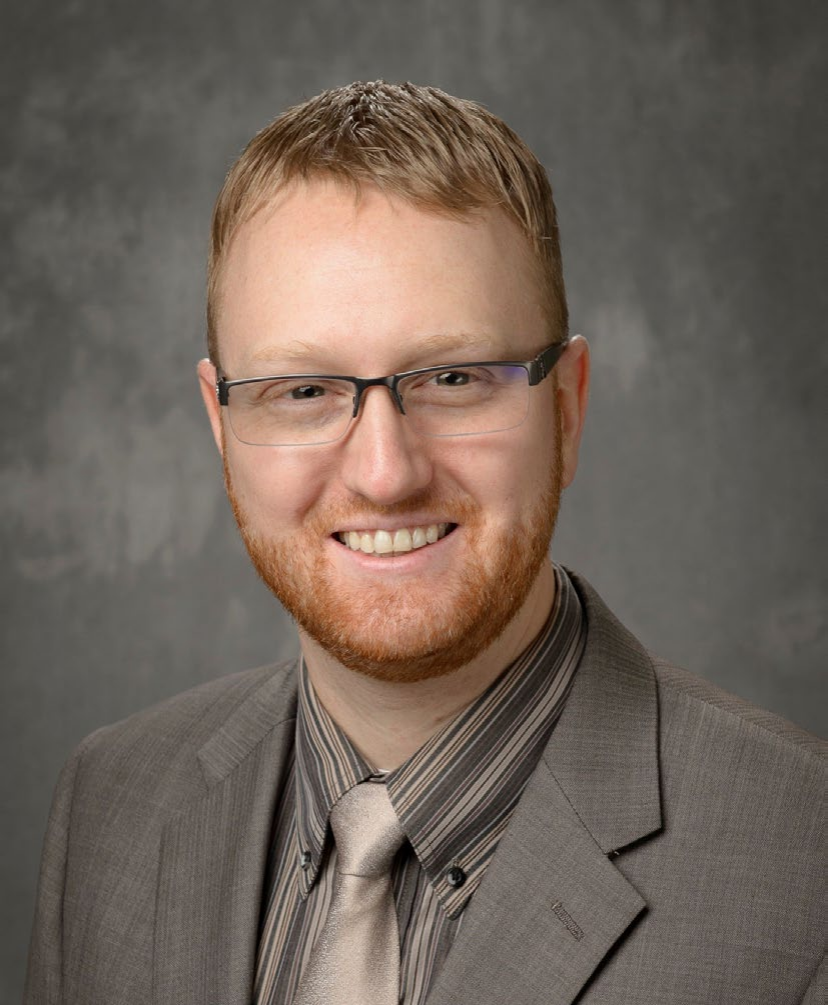

### What does your laboratory work on?

We combine functional genomics with synthetic biology approaches to study the metabolism of redox‐active plant natural products that can be harnessed for applications relevant to agriculture and human health. We are particularly interested in specialized quinones like the notorious allelochemical juglone made by black walnut trees and the anticancer compound shikonin made by the Chinese medicinal plant *Lithospermum erythrorhizon*. What's fascinating is that beyond these plants, numerous others—spread across several discrete lineages—have convergently evolved to produce structurally‐similar quinones that fulfill various roles in plant‐biotic interactions. This suggests that quinones are very effective chemical tools used by plants to interact with other organisms, yet we know little about how they're made, deployed and used. By understanding these aspects of plant quinone biochemistry, we are working to gain better insight into chemically‐mediated plant‐biotic interactions and to guide metabolic engineering strategies for producing novel quinone‐based natural products, like herbicides and chemotherapeutics.

### Tell us about the key findings from your recent article in *Plant Direct*

We report—to the best of our knowledge—the first observation of specialized quinones in nectar and their antifungal activities against common nectar fungi. The species we studied, *Impatiens glandulifera*, is known to produce specialized quinones. Interestingly, one of these compounds, 2‐methoxy‐1,4‐naphthoquinone, is considered an allelochemical that has contributed the invasion success of *I. glandulifera* in certain parts of Europe and North America. The significance of our study is that we have uncovered evidence to suggest that quinones made by *I. glandulifera*, and perhaps other flowering plants, may play an additional unsuspected role in influencing the composition of nectar microbial communities. The other finding from our study that we're really excited about is the detection of quinones in the nectar excreted by *I. glandulifera* extra‐floral nectaries. Quinones made by *I. glandulifera* have been shown by other groups to be leached via rainfall from aerial parts of the plant into the rhizosphere, although the route by which the compounds first reach the plant surface has remained a mystery. It now appears that extra‐floral nectaries may be the conduit by which quinones are deployed from vegetative organs of *I. glandulifera*.

### How did you get the idea to do this study?

Honestly, it was somewhat serendipitous. I started as an Assistant Professor at Purdue in 2016. As soon as I opened the doors to my laboratory I purchased an HPLC and hired a fantastic laboratory manager, Elena (Lena) Yakubova. While Lena and I were setting up the laboratory, we were growing several quinone‐producing species and systematically profiling various organs from each to establish what plant systems we wanted to work with for studying specialized plant quinone metabolism. At the time I didn't really know anything about *I. glandulifera*. As we grew it, I started noticing these extraordinary “pin‐shaped” organs growing out of the leaf nodes and along the leaf edges. I have to admit I had no clue what they were, so I asked one of my new horticulture colleagues who told me they were extra‐floral nectaries. Intrigued, I decided to profile them and to my surprise they contained very large pool sizes of quinones. It just so happened that at the time I was preparing a review article on specialized quinone metabolism and functions. I had just come across a paper reporting that structurally‐similar quinones secreted into the pitcher traps of carnivorous *Nepenthes spp*. function as antifungal compounds to protect the digestive fluids. That's when I started wondering whether the quinones I was detecting in *I. glandulifera* nectaries might have an analogous role in nectar. To explore this further, I contacted Dr. Anna Block (USDA‐ARS‐CMAVE) who I knew from my time at the University of Nebraska‐Lincoln when I was a graduate student and she was a postdoc. Anna works on various aspects of plant chemical defense and I knew she could help us test the quinones we detected against common nectar microbes.

### What do you plan to do next, based on these current findings?

I think there are some pretty obvious follow‐ups to this study, including determining the microbial composition of *I. glandulifera* nectaries and then assessing how the reported quinones, both individually and synergistically, affects microbe growth. I think it would also be interesting and definitely worthwhile to further explore the role that the extra‐floral nectaries play in releasing allelopathic quinones into the environment. To really get at these questions, however, we need to work with *I. glandulifera* out in the field where they can interact with pollinators, microbes and other plants. Unfortunately, *I. glandulifera* has a propensity to spread due to its exploding seed capsules. I'm extremely hesitant to set up my own field experiments with *I. glandulifera* in an uncontrolled environment as I don't want to be responsible for it escaping into the area. I'm not aware of any existing nearby populations either (which is a good thing!), but there are known populations in neighboring Michigan where *I. glandulifera* is on the invasive species watchlist. As my laboratory becomes more established, I will look for opportunities and funding to study existing populations in other areas in order to get at some of the questions that have emerged from our work.

### Why did you choose to publish in *Plant Direct*?

As I alluded to above, we've had to move on from this project for now. We obviously didn't finish the whole story yet, but I felt we did complete a sound study and made noteworthy observations that could be valuable to others working with *I. glandulifera*, or with allelopathy or nectar chemistry more broadly. Rather than sit on our findings until we could revisit the work, I decided to submit to *Plant Direct*. I first considered *Plant Direct* because it is open access and visible within the plant science community, especially to members of ASPB and SEB. Indeed, after I looked through the Table of Contents I saw that several highly visible scientists in the society communities are publishing in *Plant Direct*. *Plant Direct* was also particularly attractive because it is a peer‐reviewed journal. As an Assistant Professor I need to publish, and it is imperative that my work be critically evaluated by others in the community. So, for all these reasons I decided that *Plant Direct* was the right forum to disseminate our work.

READ THE STUDY

Block, A. K.
, 
Yakubova, E.
, & 
Widhalm, J. R.
 (2019). Specialized naphthoquinones present in *Impatiens glandulifera*nectaries inhibit the growth of fungal nectar microbes. Plant Direct, 3, 1–7. 10.1002/pld3.132
PMC658954231245775
